# Intimate Partner Violence Circumstances for Fatal Violence in the US

**DOI:** 10.1001/jamanetworkopen.2023.12768

**Published:** 2023-05-10

**Authors:** Julie M. Kafka, Kathryn E. Moracco, Laurie M. Graham, Millan A. AbiNader, Mike Dolan Fliss, Ali Rowhani-Rahbar

**Affiliations:** 1 Now with Firearm Injury and Policy Research Program, University of Washington, Seattle; 2 Department of Health Behavior, University of North Carolina Gillings School of Global Public Health, Chapel Hill; 3 University of North Carolina Injury Prevention Research Center, Chapel Hill; 4 School of Social Work, University of Maryland, Baltimore; 5 School of Social Policy and Practice, University of Pennsylvania, Philadelphia; 6 Department of Epidemiology, University of Washington, Seattle; 7 Firearm Injury and Policy Research Program, University of Washington, Seattle

## Abstract

This cross-sectional study investigates intimate partner violence circumstances associated with violent deaths in the US from 2015 to 2019.

## Introduction

Intimate partner violence (IPV) can be lethal, although the total contribution of IPV to fatal violence in the US remains unknown.^1^ Researchers often study intimate partner homicide (IPH) but may overlook other IPV-related deaths. Family, children, or new dating partners can be killed in corollary homicides. A perpetrator of IPV may be killed as a result of law enforcement response to IPV (ie, legal intervention) or may die by suicide after committing homicide (ie, homicide-suicide). Additionally, IPV could contribute to suicide in the absence of other fatalities (ie, single suicide). Emerging research suggests that single suicides were more common among perpetrators of IPV than among survivors of IPV, but single suicides occurred in both groups.^2^ We sought to provide the most complete assessment available of IPV's contribution to the burden of violent deaths in the US.

## Methods

The University of North Carolina at Chapel Hill determined that this cross-sectional study was not human participant research and so was exempt from ethical review and informed consent. Our analytical process adhered to the STROBE reporting guideline.

Most US states collect data for the National Violent Death Reporting System (NVDRS). Information from death certificates and investigation reports is recorded using close-ended fields and textual summaries called death narratives. We examined intentional violent deaths in NVDRS (2015-2019) with known circumstances and available biological sex data (Figure).

**Figure.**
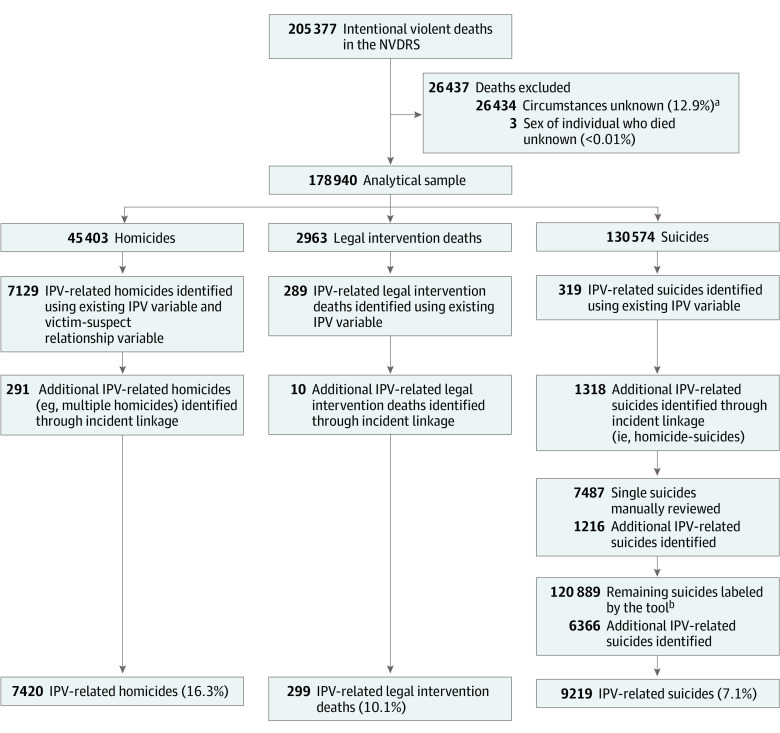
Process for Identifying Intimate Partner Violence Circumstances IPV-related fatalities do not include mercy killings, which occur when individuals are killed at their request out of compassion in order to end their pain or distress, per the National Violent Death Reporting System (NVDRS). Thus, homicides recorded as mercy killings were recorded as IPV circumstances = no. Percentages show the proportion of IPV-related deaths for each death manner. IPV indicates intimate partner violence. ^a^The Centers for Disease Control and Prevention advises excluding deaths in NVDRS with unknown circumstances from analyses of common precursors for fatal violence. Records provide missing circumstances information only if details about the death were never determined or if reporting agencies chose not to share circumstantial details with NVDRS. ^b^The supervised machine learning tool was not applied to suicides associated with a homicide. Accepted methods exist for detecting IPV circumstances in homicide-suicide events (ie, by incident identifier).

In NVDRS, IPV circumstances (yes or no) are systematically recorded for homicides and legal intervention deaths but not suicides.^3^ This lack of documentation constitutes a barrier for research. To address this limitation, we used a multipronged approach to record IPV circumstances (Figure). We first verified IPH and corollary deaths using the NVDRS IPV and victim-suspect relationship variables. Next, we linked deaths by incident identifier so that IPV circumstances applied to all deaths from the same event (eg, homicide-suicides and multiple homicides). For remaining suicides, we used novel methods, including incorporating data from prior hand-reviewed NVDRS death narratives^4,5^ and using a validated supervised machine learning tool. This tool uses a dictionary approach with natural language processing to extract keywords and phrases from death narrative text. Vectorized keywords and phrases are then fed into a random forest model that labels IPV circumstances for each suicide (yes or no) with robust sensitivity (0.70) and specificity (0.98).^6^

## Results

Among 178 940 intentional violent deaths (139 143 deaths among males and 39 797 deaths among females), 9.5% had IPV circumstances. The most common IPV-related fatality was single suicide (most common among males) (Table), followed by IPH (most common among females). Among deaths with IPV circumstances, 64.5% involved a firearm. We identified 55.2% of IPV-related fatalities using conventional methods and 44.8% using novel methods.

**Table.** Characteristics of IPV-Related Deaths

Characteristic	IPV-related deaths, No. (%)^a^		
	Overall (N = 16 938)	Females (n = 6085)^b^	Males (n = 10 853)^b^
Death type			
IPH	5198 (30.7)	3937 (64.7)	1261 (11.6)
Corollary homicide	2222 (13.1)	552 (9.1)	1670 (15.4)
Legal intervention	299 (1.8)	8 (0.1)	291 (2.7)
Suicide after homicide	1529 (9.0)	86 (1.4)	1443 (13.3)
Single suicide	7670 (45.3)	1495 (24.6)	6175 (56.9)
Other suicide type^c^	20 (0.1)	7 (0.1)	13 (0.1)
IPV circumstances identification method			
Conventional			
NVDRS IPV variable^d^	7737 (45.7)	4384 (72.0)	3353 (30.9)
Incident linkage	1619 (9.6)	214 (3.5)	1405 (12.9)
Novel			
Hand-coded suicide death narrative	1216 (7.2)	246 (4.0)	970 (8.9)
Supervised machine learning tool	6366 (37.6)	1241 (20.4)	5125 (47.2)
Age, y			
<10	161 (1.0)	92 (1.5)	69 (0.6)
10-24	2220 (13.1)	897 (14.7)	1323 (12.2)
25-40	6889 (40.7)	2452 (40.3)	4437 (40.9)
41-54	4641 (27.4)	1649 (27.1)	2992 (27.6)
55-70	2338 (13.8)	746 (12.3)	1592 (14.7)
≥71	689 (4.1)	249 (4.1)	440 (4.1)
Weapon or method			
Firearm	10928 (64.5)	3469 (57.0)	7459 (68.7)
Strangling, hanging, or suffocation	2593 (15.3)	850 (14.0)	1743 (16.1)
Poisoning	668 (3.9)	322 (5.3)	346 (3.2)
Sharp instrument	1568 (9.3)	773 (12.7)	795 (7.3)
Other	1181 (7.0)	671 (11.0)	510 (4.7)

Abbreviations: IPH, intimate partner homicide; IPV, intimate partner violence; NVDRS, National Violent Death Reporting System.

^a^ There were 178 940 total deaths overall (9.5% IPV related), 39 797 total deaths among women (15.3% IPV related), and 139 143 total deaths among men (7.8% IPV related).

^b^ Sex is recorded based on the death certificate and may not accurately reflect the person’s gender identity. Information about gender identity is considered unreliable and largely missing in NVDRS.

^c^ Includes suicides that were connected to other suicide events (eg, suicide pacts) or connected to a death of undetermined intent.

^d^ Includes an additional 17 intimate partner homicides that were separately identified using the victim-suspect relationship variable.

## Discussion

This cross-sectional study found that IPV contributed to more violent deaths in the US than previously reported; nearly 1 in 10 violent deaths had IPV circumstances. While IPV was associated with sex, findings underscore the need to engage males and females to prevent IPV while addressing shared underlying risk factors associated with IPV, homicide, and suicide. Most IPV-related fatalities involved a firearm, highlighting the importance of implementing and enforcing firearm restrictions for perpetrators of IPV. More resources may also be needed to help survivors of IPV and their families interrupt IPV before it can contribute to a fatality.

Our study has some limitations. IPV would be recorded in NVDRS only if death investigators learned about IPV and felt it was a salient factor associated with the death. In particular, coercive control or more historical IPV may be undercounted.^2^ Thus, our findings likely underestimate the contribution of IPV to violent fatalities. NVDRS data are also not yet nationally representative, and there were limited details about prior IPV experiences (ie, surviving vs perpetrating IPV) among individuals who died. Future research should examine how different experiences or roles in an abusive relationship may be differentially associated with risk for violent death in living populations.

## Notes

Supplement.
Data Sharing Statement
