# Supplementary material for: Intimate Partner Violence Circumstances for Fatal Violence in the US
Source: JAMA Netw Open. 2023 May 10;6(5):e2312768. doi: 10.1001/jamanetworkopen.2023.12768 (PMC10173018; doi:10.1001/jamanetworkopen.2023.12768)
Supplement: Supplement. — Data Sharing Statement [file jamanetwopen-e2312768-s001.pdf]

Data Sharing Statement

Kafka. Intimate Partner Violence Circumstances for Fatal Violence in the US. *JAMA Netw Open*. Published May 10, 2023. doi:10.1001/jamanetworkopen.2023.12768

Data
Data available: No

Additional Information
Explanation for why data not available: The Restricted Access Data file (RAD) from the National Violent Death Reporting System (NVDRS) can be obtained through a request to the Centers for Disease Control and Prevention (CDC). The supervised machine learning tool used in this study is available online, along with a tutorial at the following link: https://github.com/jkafka/IPVsuicide.
